# Polyethylene Microplastics Exert Dose-Dependent Effects on the Growth, Physiology, and Rhizosphere Microbiome of *Persicaria capitata*

**DOI:** 10.3390/biology15070573

**Published:** 2026-04-02

**Authors:** Rou Ma, Ying Liu, Ruhuan Wang, Suhang Li, Qiong Yang, Yue Chen, Jun Ren, Yang Luo, Yangzhou Xiang, Xuqiang Luo

**Affiliations:** 1 School of Geography and Resources, Guizhou Education University, Guiyang 550018, China; 2 School of Biological Sciences, Guizhou Education University, Guiyang 550018, China

**Keywords:** polyethylene microplastics, *Persicaria capitata*, antioxidant enzymes, microbial community, dose effect

## Abstract

Microplastic pollution has become a major environmental challenge for terrestrial ecosystems; however, the mechanisms by which it affects the growth of medicinal plants and their rhizosphere microecology remain unclear. This study selected the important medicinal herb *P. capitata* as the research subject. A pot experiment was conducted to investigate the effects of different concentrations (0%, 1%, 4%, and 8% *w*/*w*) of polyethylene microplastics (PE-MPs) on its growth performance, physiological responses, and rhizosphere bacterial communities. The main findings are as follows: (1) PE-MPs exhibited a hormetic effect on the biomass of *P. capitata*. A low concentration (1%) slightly promoted total biomass (44.78 g) compared to the control (40.94 g), whereas higher concentrations caused significant inhibition. Total biomass decreased by 28.5% and 53.5% under the 4% and 8% treatments, respectively, indicating that the transition from stimulation to inhibition occurred between the 1% and 4% PE-MPs. (2) Chlorophyll a was more sensitive to stress, with its content significantly increasing under the 1% treatment but declining at higher concentrations. (3) Regarding the antioxidant system, POD activity was significantly inhibited at low and medium concentrations, while MDA content increased significantly only under the 8% treatment (by 72%). SOD and CAT showed no significant changes, indicating that POD and MDA were more sensitive indicators of oxidative damage. (4) PE-MPs significantly reduced rhizosphere bacterial community richness (Chao1 index), and the loss of microbial diversity was highly coupled with the decline in plant biomass and the exacerbation of oxidative damage. In conclusion, this study confirms a threshold effect in the toxicity of PE-MPs to *P. capitata*, with the transition from hormetic stimulation to toxic inhibition occurring between 1% and 4% (*w*/*w*). Furthermore, rhizosphere microecological imbalance is identified as a key indirect mechanism underlying phytotoxicity. These findings provide a new theoretical basis for understanding the potential impacts of microplastic pollution on medicinal plants and agroecosystems.

## Simple Summary

Microplastic pollution is an emerging concern in agricultural systems, yet its effects on medicinal plants remain poorly understood. In this study, we investigated the impacts of polyethylene microplastics, a common component of agricultural plastic films, on the growth and health of *Persicaria capitata* (*P. capitata*), an important medicinal herb. Our results showed that low concentrations of microplastics slightly promoted plant growth, whereas higher concentrations caused significant harm, reducing plant size by over 50% and impairing photosynthetic pigments. Microplastic exposure also disrupted beneficial bacterial communities in the rhizosphere and induced stress responses in plant cells. These findings indicate that microplastics can indirectly affect plants by disturbing soil microbial ecosystems. This study highlights a previously overlooked threat to the cultivation of medicinal plants and the long-term sustainability of agricultural soils, underscoring the need for careful management of plastic use in farming.

## 1. Introduction

One of the defining environmental features of the Anthropocene is the widespread accumulation of synthetic polymers in ecosystems, with plastic pollution emerging as a critical ecological concern [1,2,3]. Over the past few decades, global plastic production has surged, exceeding 390 million tons annually. A substantial proportion of this plastic inevitably enters terrestrial ecosystems through pathways such as the fragmentation of agricultural mulch film, the application of sewage sludge to farmland, and atmospheric deposition [4,5,6,7]. Once in the soil, large plastic debris undergoes physical, chemical, and biological weathering, gradually forming microplastics (MPs), which are particles smaller than 5 mm in diameter [8,9]. Due to frequent anthropogenic disturbance, agricultural soils have been identified as a major long-term sink for microplastics, with concentrations ranging from a few particles to several thousand particles per kilogram, depending on land use patterns and the intensity of human input [10,11]. Compared to microplastics in marine environments, those in soils exhibit greater heterogeneity in polymer type (e.g., polyethylene, polypropylene, polystyrene), morphology (fibers, fragments, films), and associated chemical additives, which significantly complicates their ecological risk assessment [12,13,14]. Consequently, unraveling the impacts of microplastics on soil health and agricultural productivity has become a central theme in contemporary environmental science research [15].

A growing body of evidence indicates that the accumulation of microplastics in soil can profoundly affect plant growth, although reported results are often inconsistent and highly context-dependent [16,17]. The phytotoxicity of microplastics is typically attributed to both direct and indirect mechanisms [18]. Direct effects may involve the physical blockage of seed pores, the internalization of particles within root cells (particularly for nanoplastics), and the release of toxic monomers or plasticizers such as phthalates and bisphenol A [19,20]. Indirect effects manifest through the ability of microplastics to alter fundamental soil physicochemical properties, including bulk density, water-holding capacity, and aggregate stability, thereby influencing root architecture and nutrient acquisition efficiency [21,22]. Furthermore, microplastics can modulate the bioavailability of essential nutrients and pollutants, potentially exacerbating or alleviating plant stress [23]. For instance, studies on major crops have shown that polystyrene microplastics can trigger oxidative bursts and photosynthetic inhibition in wheat [24,25], while PE-MPs have been demonstrated to impair root elongation and cause genetic damage in rice [26]. However, contradictory findings have also been reported, with low concentrations of certain microplastics stimulating plant biomass accumulation [27,28]. These conflicting conclusions underscore the urgent need for systematic dose–response studies to clarify the thresholds and mechanistic pathways of microplastic-induced phytotoxicity.

Beyond plant physiological responses, the rhizosphere, as a hotspot for plant-microbe interactions, serves as a critical interface where microplastics exert their indirect effects. Soil microorganisms are highly sensitive to environmental disturbances, and the introduction of microplastics can act as a selective pressure, reshaping the composition, diversity, and functional potential of rhizosphere bacterial communities [29]. Subsequent alterations in the structure of the rhizosphere microbial community can have cascading effects on plant health by modulating nutrient cycling (e.g., nitrogen fixation, phosphorus solubilization), the synthesis of plant hormones, and the suppression of soilborne pathogens [30,31]. Recent metagenomic analyses have revealed that PE-MPs can enrich specific microbial taxa with plastic-degrading potential while simultaneously reducing the abundance of beneficial symbionts, such as arbuscular mycorrhizal fungi [32]. For example, a study by Lu et al. [33] found that PE-MPs significantly altered the soil microbiome, leading to shifts in community assembly processes and reducing the complexity of microbial interaction networks. Similarly, Lai et al. [34] discovered that the presence of microplastics disrupts the coupling between plant functional traits and microbial diversity in agroecosystems. Despite these advances, most current research remains focused on food crops like rice and wheat, with limited understanding of the responses of medicinal or native plants. These plants often possess unique physiological traits and ecological adaptation strategies, and how they respond to microplastic pollution represents a significant knowledge gap.

Although research on microplastic-plant interactions is rapidly accumulating, several critical scientific gaps persist, hindering a comprehensive understanding of their ecological effects. First, few studies have simultaneously integrated plant phenotypic responses (biomass allocation), physiological and biochemical adjustments (photosynthetic pigment dynamics and antioxidant defense mechanisms), and belowground microbial community succession within a single experimental framework [35]. Such an integrative approach is essential for establishing causal links between microplastic-induced soil perturbations and aboveground plant performance. Second, the concentrations tested in many experiments often far exceed environmentally relevant levels, making it difficult to extrapolate results to field conditions [11,36]. For instance, while high doses (e.g., >5% *w*/*w*) of microplastics unequivocally demonstrate toxicity, the subtle changes occurring at more environmentally relevant concentrations (e.g., 0.1–2% *w*/*w*), which may hold significant long-term ecological implications, are frequently overlooked [37]. Third, the plant species investigated to date are taxonomically limited. *Persicaria capitata* (Buch.-Ham. ex D. Don) H.Gross (*P. capitata*), an important medicinal herb widely distributed in the unique karst regions of Southwest China, often faces specific anthropogenic disturbances. However, to date, no studies have reported on the impact of microplastics on this species. Incorporating such non-model species into research frameworks is crucial for assessing microplastic pollution in biodiversity hotspots.

To address these research gaps, we designed a pot experiment aimed at systematically investigating the dose-dependent effects of PE-MPs on the growth performance, physiological metabolism, and rhizosphere microbial ecology of *P. capitata*. Polyethylene was chosen as the test polymer because it is the dominant type in agricultural soil pollution, being a primary component of mulch film residues [38]. The experiment established four concentration gradients (0%, 1%, 4%, and 8% *w*/*w*) designed to encompass both environmentally relevant levels and extreme exposure scenarios, thereby capturing potential hormetic effects and toxicity thresholds. This study proposed the following hypotheses: (i) Low-dose PE-MP exposure would induce a hormetic effect, stimulating plant growth and pigment accumulation, whereas high-dose exposure would trigger oxidative stress and inhibit biomass production; (ii) PE-MPs would alter the activities of key antioxidant enzymes (superoxide dismutase [SOD], peroxidase [POD], catalase [CAT]) in a concentration-dependent manner and increase the content of the lipid peroxidation product malondialdehyde (MDA); (iii) Changes in plant physiological status would be closely linked to alterations in the diversity and composition of rhizosphere bacterial communities, with the latter being revealed through high-throughput sequencing of the 16S rRNA gene. By integrating plant ecophysiology with microbial ecology, this study aims to provide a mechanistic understanding of how PE-MPs affect the soil-microbe-plant continuum, thereby offering a scientific reference for the precise ecological risk assessment of plastic pollution in terrestrial ecosystems.

## 2. Materials and Methods

### 2.1. Experimental Materials

The topsoil (0–20 cm) used in this experiment was collected from an upland in Wudang District, Guiyang City, Guizhou Province. This region is characterized by karst topography and has been subjected to long-term conventional agricultural practices, including the historical use of plastic mulch films, application of organic amendments (e.g., sewage sludge or farmyard manure), and mechanical tillage. These activities represent typical anthropogenic pressures in the area and are recognized as potential sources of microplastic accumulation in agricultural soils. However, compared to industrial or landfill sites, this location represents a moderately impacted agricultural background, suitable for assessing the effects of additional microplastic spiking. After removing visible stones and organic debris, the soil was air-dried and passed through a 2 mm sieve for subsequent analysis. Its basic physicochemical properties were as follows: pH 6.12, organic matter 28.19 g kg^−1^, alkali-hydrolysable nitrogen 25.06 mg kg^−1^, and available phosphorus 31.91 mg kg^−1^. The background microplastic concentration in collected soil was not quantified prior to the experiment due to methodological challenges in extracting small particles (<150 μm) and the study’s primary objective of establishing dose–response relationships. Even if present, background levels in agricultural soils typically (<0.1% *w*/*w*) are orders of magnitude lower than our lowest treatment (1%, 10,000 mg/kg). Therefore, added PE-MPs are considered the dominant factor driving observed effects. The experimental materials included PE-MPs (density 0.92 g cm^−3^, particle size ≈ 150 μm) purchased from Guangdong Suhua Powder Material Co., Ltd. (Dongguan, China), and high-quality *P. capitata* seeds (with pure color and plump grains) obtained from Guizhou Xingqian Technology Development Co., Ltd. (Guiyang, China). These PE-MPs were spherical beads with a uniform nominal diameter. Prior to use, they were washed with 75% ethanol for 30 min, followed by three rinses with sterilized purified water, and then air-dried to remove any potential surface contaminants from the manufacturing process. The plants were cultivated in plastic pots (top diameter 20 cm, bottom diameter 18 cm, height 12 cm).

### 2.2. Experimental Design

The experiment was conducted under natural light and temperature conditions in a plastic greenhouse located at Guizhou Education University, Guiyang City, Guizhou Province, China (106°47′ E, 26°38′ N). A completely randomized design with one factor was used. The factor was the mass percentage of PE-MPs added to the soil, which included four levels: 0% (control), 1%, 4%, and 8%. These percentages corresponded to additional amounts of 0, 10, 40, and 80 g of PE microplastics per pot, respectively. Each treatment was replicated three times, resulting in a total of 12 experimental units. In this study, PE-MPs were added based on mass percentage (%, *w*/*w*) to establish concentration gradients, a widely adopted approach in dose–response studies. While this method is appropriate for comparative toxicity assessment, we acknowledge that particle shape and specific surface area can influence microplastic effects on soil structure. The spherical morphology and uniform size of the PE-MPs used here partially control for surface area variability across treatments. However, surface area-based dosage may provide complementary mechanistic insight in future studies.

For each experimental unit, 1000 g of air-dried and sieved (<2 mm) soil was weighed. The pre-determined amount of PE microplastics for each treatment was then added to the soil. The soil–microplastic mixture was homogenized thoroughly to ensure even distribution before being transferred to a labeled plastic pot. After filling, the soil in each pot was moistened with tap water to 60% of its field capacity and allowed to equilibrate for two days to stabilize the microplastic-soil interaction. On the sowing day, *P. capitata* seeds were surface sterilized by immersion in 75% ethanol for 30 min, followed by multiple rinses with purified water. The sterilized seeds were then soaked in purified water for 12 h to promote germination. Subsequently, ten uniformly sized seeds were sown at a depth of 1–2 cm in each pot. Throughout the growth period, plants were managed under consistent conditions. Irrigation was applied as needed based on ambient temperature and soil moisture observations to maintain optimal plant growth and avoid water stress.

The selected concentration gradient reflects a range of environmental relevance and mechanistic exploration. Environmentally relevant concentrations of microplastics in agricultural soils typically range from <0.01% to 0.5% *w*/*w*, with values exceeding 1% *w*/*w* documented only in hotspot areas such as long-term mulched fields or sites receiving repeated sludge applications. Accordingly, the 1% treatment approximates an environmentally relevant upper bound, while the 4% and 8% treatments serve as stress-test concentrations designed to establish complete dose–response curves, identify toxicity thresholds, and amplify mechanistic signals that may be subtle at lower concentrations.

### 2.3. Sample Collection and Analysis

It is important to clarify that in this study, microplastics were introduced as an experimental treatment factor (independent variable) rather than being extracted and quantified as an analyte (dependent variable) post-experiment. The experimental design involved spiking soil with known concentrations of pristine PE-MPs to establish pollution gradients. Soil samples collected at 90 DAS were therefore processed for physicochemical analysis and microbial community characterization, not for the purpose of microplastic extraction or quantification. The core endpoints of this study are the responses of plant growth, physiological parameters, and rhizosphere bacterial communities to the defined PE-MP treatments. At 90 days after sowing (DAS), when plants reached a key growth stage, both soil and plant samples were collected from each pot for laboratory analysis. Plant samples were carefully separated into roots and shoots, and soil samples were homogenized for further physicochemical and biological assays.

#### 2.3.1. Measurement of Growth Parameters

After 90 days of growth, the plants were carefully harvested. The shoots of *P. capitata* were cut off close to the soil surface using scissors. The soil was then loosened, and the roots were separated and collected. A brush was used to remove any adhering soil. The shoots and roots were thoroughly rinsed with tap water, and surface moisture was blotted dry with absorbent paper. The fresh weight of each part was recorded to determine the above-ground and below-ground biomass. Total plant biomass was calculated as the sum of these two components. The root-to-shoot ratio was determined by dividing below-ground biomass by above-ground biomass.

#### 2.3.2. Determination of Chlorophyll Content

According to the method of Wintermans and De Mots (1965) [39], fresh leaf tissue (0.2 g) was ground in an ice bath with a small amount of quartz sand, calcium carbonate powder, and 2–3 mL of pre-chilled 95% ethanol until the tissue turned white. The homogenate was allowed to stand for 3 min, then filtered, and the residue was rinsed with the same ethanol solution to a final volume of 25 mL. The absorbance of the extract was measured using a spectrophotometer at 665 nm and 649 nm. The contents of chlorophyll a (mg g^−1^), chlorophyll b (mg g^−1^), total chlorophyll (mg g^−1^), and the ratio of chlorophyll a/b were calculated according to Formulas (1), (2), (3), and (4), respectively:

Chlorophyll a = (13.95 × D_665_ − 6.80 × D_649_) × V/(W × 1000) (1)

Chlorophyll b = (24.96× D_649_ − 7.32 × D_665_) × V/(W × 1000) (2)

Total Chlorophyll = Chlorophyll a + Chlorophyll b(3)

Chlorophyll a/b = Chlorophyll a ÷ Chlorophyll b(4)

where D665 and D649 are the absorbance values of the sample solution at 665 nm and 649 nm, respectively; V is the volume of the extraction solution (mL); W is the sample mass (g).

#### 2.3.3. Determination of Antioxidant Enzyme Activities

*P. capitata* were collected to determine the activities of superoxide dismutase (SOD), peroxidase (POD), and catalase (CAT), as well as the content of malondialdehyde (MDA). Specifically, (1) SOD assay: Leaf tissue (0.4 g) was ground with 1 mL of pre-cooled extraction buffer on ice. The homogenate was rinsed with an additional 3 mL of buffer, transferred to a centrifuge tube, and brought to a final volume of 4 mL. After centrifugation at 4000× *g* for 20 min at 4 °C, the supernatant was collected and kept on ice. SOD activity was then measured using a specific kit (No. R22261) according to the manufacturer’s protocol. (2) POD assay: Leaf tissue (0.5 g) was homogenized with 4 mL of ice-cold extraction buffer in an ice bath. The homogenate was subsequently centrifuged at 10,000× *g* for 20 min at 4 °C, and the resulting supernatant was stored at −20 °C for later analysis. POD activity was determined using a commercial assay kit (No. R30312, Shanghai Yuanye Bio-Technology Co., Ltd., Shanghai, China) following the manufacturer’s instructions. (3) CAT assay: Fresh tissue (0.5 g) was homogenized with 2 mL pre-cooled buffer (No. R22073^−1^00T kit), then adjusted to 10 mL. After 10 min at 4 °C, it was centrifuged at 1200 r/min for 30 min at 4 °C, and the supernatant was collected. For measurement, 0.2 mL sample was mixed with 1.5 mL buffer and 1.0 mL distilled water, pre-warmed to 25 °C. The reaction was initiated with 0.3 mL 100 mM H_2_O_2_. Absorbance at 240 nm was recorded every minute for four readings, and CAT activity was calculated from the decrease rate. (4) MDA assay: Leaf tissue (0.4 g) was homogenized with 4 mL of tissue homogenization buffer in an ice bath. The homogenate was then centrifuged at 4000× *g* for 10 min at 4 °C, and the supernatant was collected. Finally, MDA content was quantified using a dedicated assay kit (No. R21874) strictly following the manufacturer’s specifications.

#### 2.3.4. Rhizosphere Microbial Community

Total genomic DNA was extracted from soil samples using the CretMag Power Soil DNA Kit (Cretaceous, Suzhou, China) following the manufacturer’s instructions. DNA integrity was assessed by 1% agarose gel electrophoresis, and DNA concentration and purity were determined using a NanoDrop2000 spectrophotometer (Allsheng, Hangzhou, China). The V3-V4 hypervariable region of the bacterial 16S rRNA gene was amplified by PCR using primers 341F (5′-CCTAYGGGRBGCASCAG-3′) and 806R (5′-GGACTACHVGGGTWTCTAAT-3′). The thermal cycling conditions were as follows: initial denaturation at 94 °C for 2 min, followed by 30 cycles of denaturation at 94 °C for 30 s, annealing at 55 °C for 30 s, and extension at 72 °C for 30 s, with a final extension at 72 °C for 10 min. PCR reactions (50 μL) contained 25 μL of 2 × ES Taq MasterMix (Dye), 2 μL of each forward and reverse primer (10 μmol·L^−1^), 10 ng of template DNA, and ddH_2_O added to reach a final volume of 50 μL. Three technical replicates were performed for each sample. PCR products were separated by 2% agarose gel electrophoresis, excised, and purified using the AxyPrep DNA Gel Extraction Kit (Axygen Biosciences, Union City, CA, USA), followed by precise quantification with a Quantus Fluorometer (Promega, Madison, WI, USA). Sequencing libraries were constructed using the NEXTflex Rapid DNA-Seq Kit (Bioo Scientific, Austin, TX, USA), which included adapter ligation, magnetic bead-based size selection to remove adapter dimers, PCR enrichment, and library recovery. After quality control, the libraries were sequenced on the Illumina NovaSeq PE250 platform (Illumina, San Diego, CA, USA) using paired-end sequencing.

#### 2.3.5. Limitations Regarding Soil Physicochemical Analysis

This study primarily focused on plant physiological responses and rhizosphere microbial community dynamics under PE-MP stress. Soil physicochemical properties, including bulk density, pH, organic matter content, and nutrient availability, were not systematically measured in this experiment. While these parameters are recognized as important mediators of plant–microbe–microplastic interactions, their exclusion constitutes a limitation of the present study. Consequently, mechanistic inferences involving soil physicochemical pathways are discussed as hypotheses supported by prior literature and indirect evidence from the present dataset, and direct causal claims are not made.

### 2.4. Data Analysis

Experimental data were preliminarily processed using Excel 2016, and results were expressed as mean ± standard deviation (n = 3). One-way analysis of variance (ANOVA) was used to assess the effects of different polyethylene microplastic (PE-MP) concentrations on the growth parameters, physiological indices, and rhizosphere bacterial community diversity of *P. capitata*. When significant differences were detected, the least significant difference (LSD) test was applied for multiple comparisons at a 95% confidence level. Graphs were generated using Origin 2026 software (OriginLab, Northampton, MA, USA). The Kruskal–Wallis rank sum test was used to compare the differences in soil bacterial community alpha diversity under different treatments. Mantel tests with 999 permutations were performed to evaluate the relationships between *P. capitata* biomass and physiological indices, soil microbial diversity under PE-MPs pollution, using the ‘LinkET’ package (https://github.com/Hy4m/linkET, accessed on 31 March 2026) [40]. All statistical analyses were performed using R statistical software (version 4.3.3; R Core Team, Vienna, Austria).

## 3. Results

### 3.1. Effects of PE-MPs on the Biomass of P. capitata

**Figure 1:**
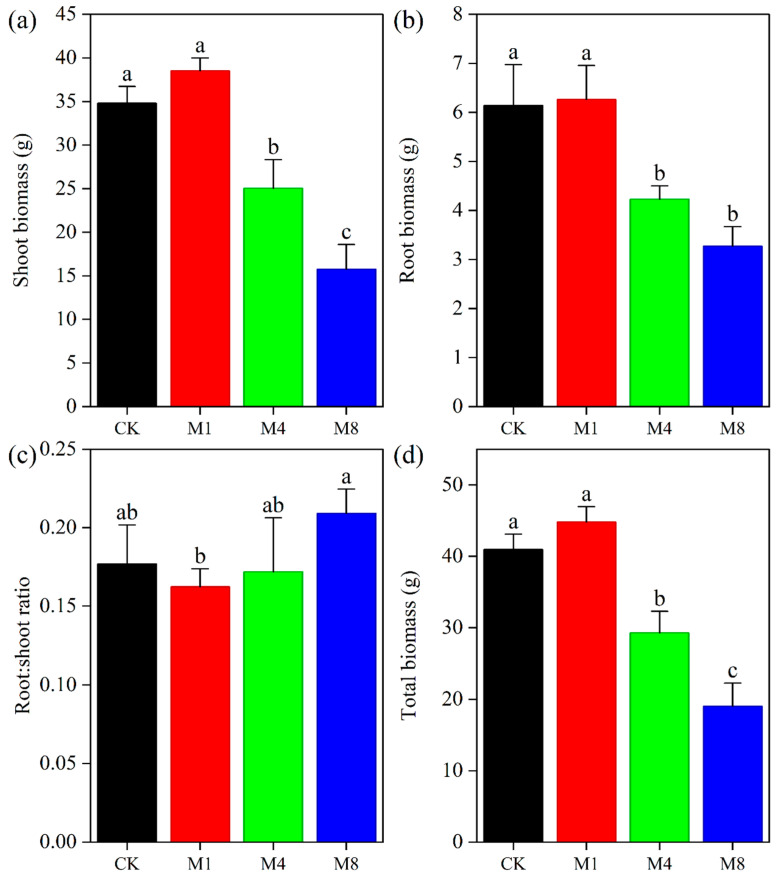
Biomass of *P. capitata* as Affected by PE-MPs. Error bars represent ± standard deviation (n = 3). Different lowercase letters indicate significant differences among treatments (*p* < 0.05). (**a**) Shoot biomass; (**b**) Root biomass; (**c**) Root:shoot ratio; (**d**) Total biomass.

The biomass accumulation of *P. capitata* exhibited a concentration-dependent response to PE-MPs stress (Figure 1). Regarding shoot biomass (Figure 1a), compared to the control (CK, 34.79 g), treatment M1 (38.52 g) resulted in a slight but non-significant increase. However, exposure to higher concentrations significantly inhibited shoot growth. The M4 treatment sharply reduced shoot biomass to 25.04 g, a significant decrease from both CK and M1, while the most severe inhibition was recorded under M8, with shoot biomass plummeting to 15.76 g, which was statistically lower than all other treatments.

A similar inhibitory trend was noted for root biomass (Figure 1b). Root biomass in the CK and M1 treatments were statistically comparable, measuring 6.14 g and 6.26 g, respectively. A significant reduction was first observed under M4 (4.23 g), and this inhibition persisted under M8 (3.27 g). Notably, the root biomass values for M4 and M8 did not differ significantly from each other, suggesting a plateau in the inhibitory effect on root growth at higher concentrations. The root-to-shoot ratio (Figure 1c) was significantly altered only at the highest concentration (M8), where it increased to 0.21, indicating a more pronounced allocation of resources to root growth under severe stress.

This shift in allocation patterns was further manifested in total biomass (Figure 1d), which integrated these organ-specific responses. Consistent with the shoot biomass trend, total biomass in the M1 treatment (44.78 g) was marginally, though not significantly, higher than in CK (40.94 g). Thereafter, total biomass declined precipitously with increasing PE-MP concentration. The M4 treatment resulted in a total biomass of 29.27 g, a significant reduction of approximately 28.5% compared to CK. The M8 treatment caused the most drastic effect, reducing total biomass to 19.03 g, which was significantly lower than all other treatment groups. These results demonstrate that high concentrations of PE-MPs (M4 and M8) exert a significant phytotoxic effect on *P. capitata*, severely impairing both shoot and root development and overall plant productivity.

### 3.2. Response of P. capitata Chlorophyll to PE-MPs

**Figure 2:**
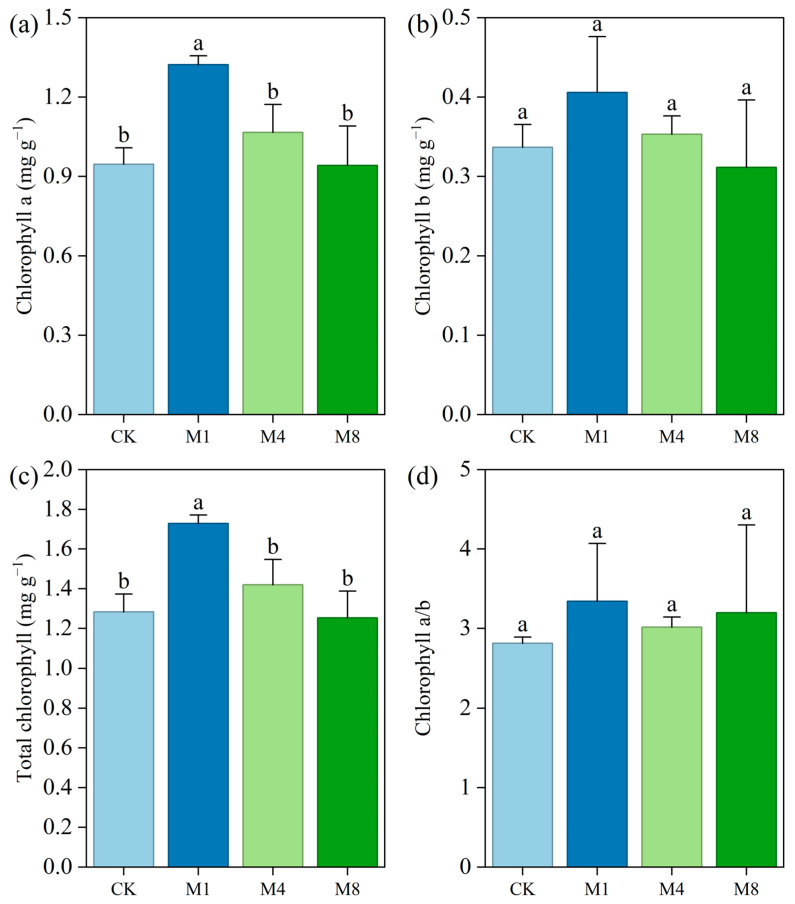
Chlorophyll content in *P. capitata* under different concentrations of PE-MPs. (**a**) Chlorophyll a, (**b**) chlorophyll b, (**c**) total chlorophyll, and (**d**) chlorophyll a/b ratio. Error bars represent standard deviation (n = 3). Different lowercase letters indicate significant differences among treatments (*p* < 0.05).

The results in this study illustrated the physiological response of *P. capitata* to varying concentrations of PE-MPs in terms of chlorophyll content (Figure 2). Under low-dose PE-MPs stress (1%), a significant increase in chlorophyll a content was observed (1.32 mg g^−1^), marking a 38.9% rise compared to the control (0.95 mg g^−1^, *p* < 0.05) (Figure 2a). Similarly, total chlorophyll content peaked at 1.73 mg g^−1^ under the same treatment (Figure 2c), significantly higher than the control group (1.28 mg g^−1^, *p* < 0.05). These results suggest a potential low-concentration stimulation effect, where moderate levels of MP stress may promote pigment accumulation, possibly as an adaptive response to maintain photosynthetic capacity under mild stress conditions. However, as MP concentration increased to 4% and 8%, chlorophyll a and total chlorophyll declined progressively, reaching levels comparable to or slightly below the control, indicating the onset of stress-induced inhibition. This dose-dependent response pattern suggests a threshold exists between 1% and 4% PE-MP concentration, beyond which photosynthetic pigments begin to exhibit stress symptoms.

In contrast, chlorophyll b content (Figure 2b) remained statistically unchanged across all treatments (*p* > 0.05), with values ranging from 0.31 to 0.41 mg g^−1^. This stability implies that chlorophyll b, which is primarily involved in light harvesting and photosystem stability, may be less sensitive to PE-MP exposure than chlorophyll a. Consequently, the chlorophyll a/b ratio (Figure 2d), though slightly elevated under MP treatments, showed no significant differences due to high variability, particularly in the 8% treatment group (SD = 1.11). The lack of statistical significance in a/b ratio further supports the notion that changes in total chlorophyll were largely driven by fluctuations in chlorophyll a, while the light-harvesting complex remained relatively stable under PE-MP stress.

Overall, these findings demonstrate that *P. capitata* exhibits a biphasic response to PE-MP stress: a transient stimulatory effect at low concentrations followed by inhibitory effects at higher concentrations. The significant decline in total chlorophyll under elevated MP levels may reflect impaired chlorophyll biosynthesis or accelerated pigment degradation, potentially linked to oxidative stress, nutrient imbalance, or physical disruption of root function induced by microplastic accumulation in the growth medium. These results provide foundational evidence for understanding the ecophysiological impacts of microplastic pollution on terrestrial plants and highlight the importance of considering dose-effect relationships in phytotoxicity assessments of emerging contaminants.

### 3.3. Modulation of P. capitata Antioxidant Enzymes by PE-MPs

**Figure 3:**
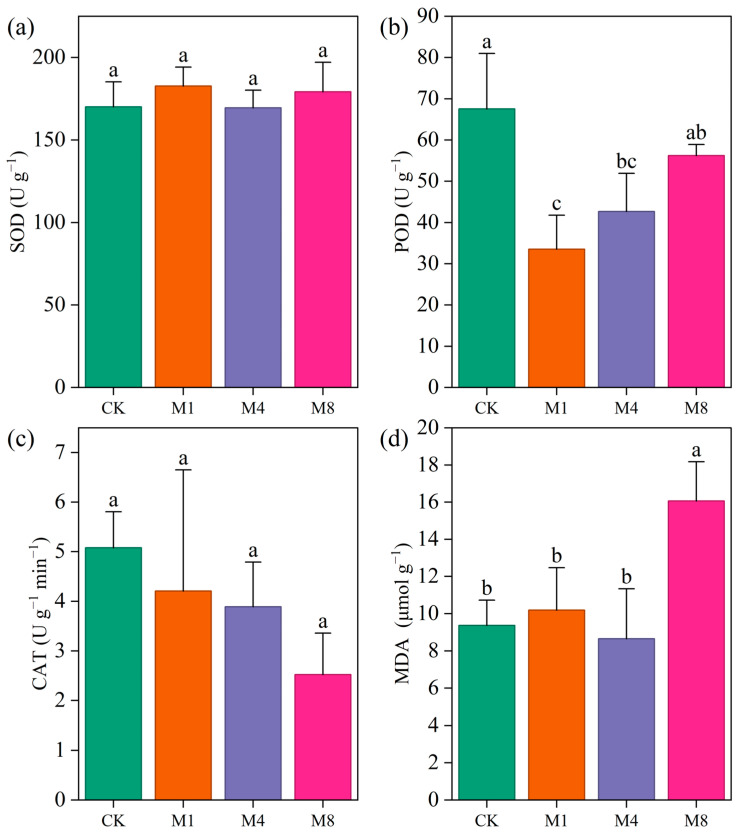
Antioxidant enzyme activities and MDA content in *P. capitata* under different concentrations of PE-MPs. (**a**) SOD activity, (**b**) POD activity, (**c**) CAT activity, and (**d**) MDA content. Error bars represent standard deviation (n = 3). Different lowercase letters indicate significant differences among treatments (*p* < 0.05).

Exposure to PE-MPs differentially modulated the antioxidant enzyme activities and lipid peroxidation levels in *P. capitata* (Figure 3). As shown in Figure 3a, superoxide dismutase (SOD) activity remained relatively stable across all treatment groups, with mean values ranging from 169.57 to 182.72 U g^−1^ FW. Statistical analysis revealed no significant differences among treatments (*p* > 0.05), indicating that SOD was not substantially responsive to PE-MP stress within the tested concentration range. In contrast, peroxidase (POD) activity exhibited a pronounced response to PE-MP exposure (Figure 3b). Compared to the control (67.56 U g^−1^ FW), POD activity significantly decreased in the M1 (33.56 U g^−1^ FW) and M4 (42.67 U g^−1^ FW) treatments, representing reductions of approximately 50% and 37%, respectively. Although POD activity in the M8 treatment (56.22 U g^−1^ FW) showed a trend of recovery toward control levels, it remained statistically comparable to both the control and intermediate treatments based on post hoc letter groupings. This non-linear, concentration-dependent pattern suggests a complex regulatory mechanism of POD in response to increasing PE-MP stress.

Catalase (CAT) activity demonstrated a gradual, though statistically non-significant, decline with increasing PE-MP concentrations (Figure 3c). Mean CAT values decreased progressively from 5.08 U g^−1^ min^−1^ in the control to 2.52 U g^−1^ min^−1^ in the highest treatment group (M8), an approximate 50% reduction. The lack of statistical significance was primarily attributable to considerable intra-group variability, particularly in the M1 treatment (SD = 2.44). Malondialdehyde (MDA) content, a biomarker of lipid peroxidation, remained relatively stable under low and moderate PE-MP stress but increased markedly at the highest exposure concentration (Figure 3d). While MDA levels in the M1 (10.19 μmol g^−1^ FW) and M4 (8.65 μmol g^−1^ FW) treatments were statistically similar to the control (9.37 μmol g^−1^ FW), the M8 treatment induced a substantial elevation to 16.06 μmol g^−1^ FW. The significant increase in MDA content at the highest PE-MP concentration suggests oxidative membrane damage. Notably, SOD and CAT showed no significant changes across treatments, while POD was significantly inhibited at low and medium concentrations, indicating a non-uniform antioxidant response. Taken together, these findings demonstrate that PE-MPs modulate the antioxidative defense system in *P. capitata* in an enzyme-specific manner, with POD activity and MDA accumulation serving as more sensitive indicators of PE-MP-induced oxidative stress compared to SOD and CAT.

### 3.4. Effects of Polyethylene Microplastics on the Diversity of Rhizosphere Soil Microbial Communities

**Figure 4:**
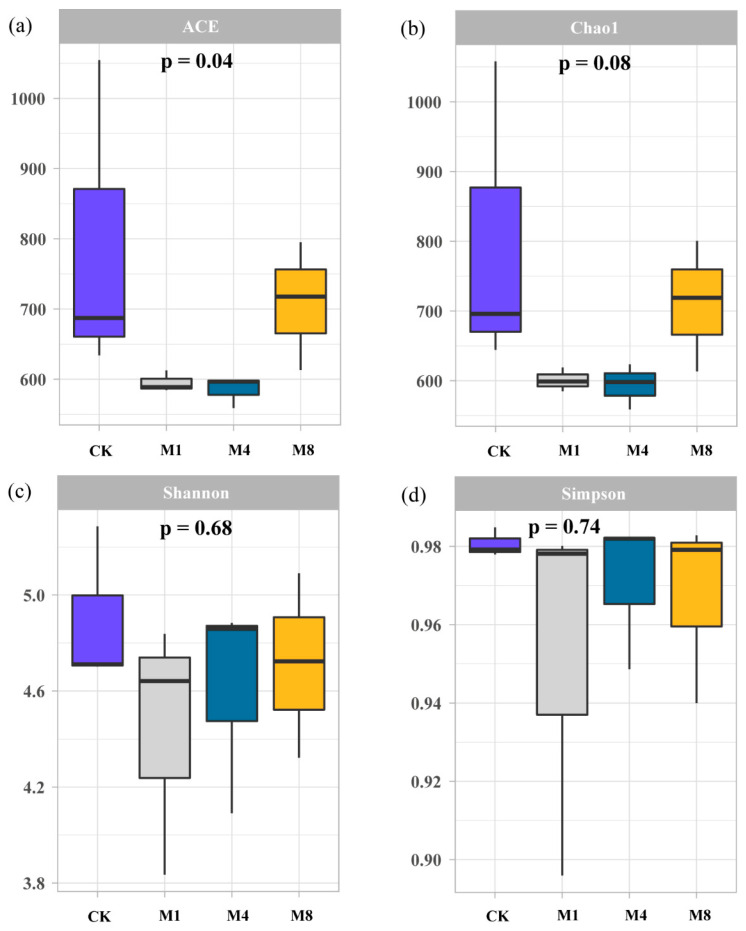
Alpha diversity of the rhizosphere soil bacterial community associated with *P. capitata* under different concentrations of PE-MPs. Box plots illustrate the median (central line), interquartile range (box boundaries), and range (whiskers) of four alpha diversity indices: (**a**) ACE richness estimator, (**b**) Chao1 richness estimator, (**c**) Shannon diversity index, and (**d**) Simpson diversity index (n = 3 per treatment group). Treatments include control (CK, no PE-MPs) and three concentrations of PE-MPs: M1 (1%), M4 (4%), and M8 (8%). Statistical comparisons among treatment groups were performed using the Kruskal–Wallis rank sum test.

The analysis revealed that PE-MP exposure significantly altered bacterial community richness (Figure 4). For the ACE index, a significant reduction was observed in the M2 treatment compared to the control (CK) (*p* < 0.05). Similarly, the Chao1 richness estimator demonstrated a marked decrease in both the M1 and M2 treatments relative to CK (*p* < 0.05), indicating that the presence of PE-MPs reduced the estimated number of operational taxonomic units (OTUs) in the rhizosphere soil. Regarding community diversity, the Shannon index was significantly lower in the M2 treatment compared to CK (*p* < 0.05), suggesting a decline in both species’ richness and evenness under moderate microplastic stress. In contrast, the Simpson index, which places greater emphasis on community evenness and the proportional abundance of dominant species, exhibited no statistically significant differences across any of the treatment groups (*p* > 0.05). This finding implies that while PE-MPs, particularly at moderate concentrations (M2), can diminish overall bacterial richness and diversity, they do not substantially disrupt the dominance structure or the relative abundance of the most prevalent taxa within the rhizosphere microbial community associated with *P. capitata*.

### 3.5. Mantel Test and Correlation Analysis

**Figure 5:**
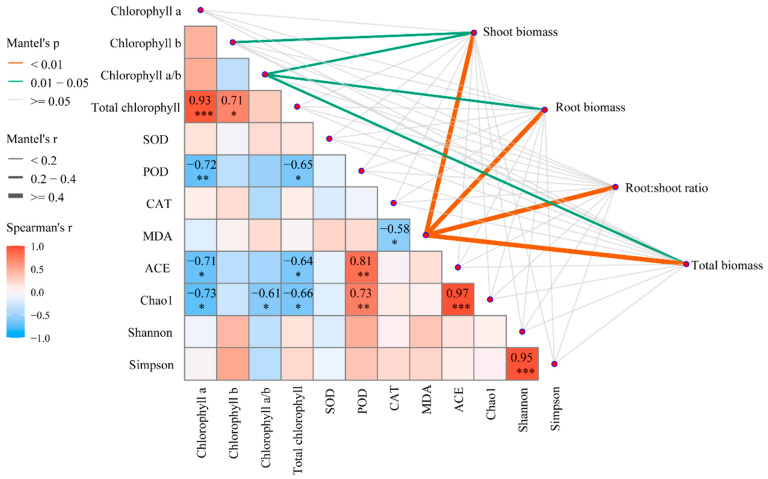
Exploratory correlation analysis of growth traits, physiological indices, and soil microbial diversity under PE-MP stress. Results are based on limited replication (n = 3 per treatment) and are intended to complement primary findings rather than establish causal relationships. Asterisks (*, **, ***) represent statistical significance at the *p* < 0.05, *p* < 0.01, and *p* < 0.001 levels, respectively.

Exploratory Mantel tests revealed consistent associations between microbial diversity indices and multiple plant functional traits, although the limited replication (n = 3 per treatment) restricts the inferential strength of these analyses (Figure 5). Plant biomass parameters showed the strongest overall association with microbial diversity (*r* ≥ 0.4, *p* < 0.01), followed by antioxidant enzyme activities (*r* = 0.2–0.4, *p* < 0.05), while chlorophyll content exhibited weaker correlations (*r* < 0.2, *p* ≥ 0.05).

Spearman’s correlation analysis further elucidated specific relationships between individual variables and microbial diversity indices. Shoot biomass demonstrated strong positive correlations with Shannon (*r* = 0.93, *p* < 0.001) and Simpson indices (*r* = 0.95, *p* < 0.001), indicating that aboveground productivity is closely linked to microbial evenness and diversity. Similarly, total biomass showed highly significant positive associations with both Shannon (*r* = 0.97, *p* < 0.001) and Simpson indices (*r* = 0.95, *p* < 0.001). In contrast, root-to-shoot ratio exhibited negative correlations with Shannon (*r* = −0.72, *p* < 0.01) and Chao1 indices (*r* = −0.71, *p* < 0.05), suggesting that under high microbial diversity conditions, plants preferentially allocate resources to aboveground growth.

Regarding antioxidant enzymes, superoxide dismutase (SOD) activity showed significant positive correlations with Shannon (*r* = 0.73, *p* < 0.01) and Simpson indices (*r* = 0.81, *p* < 0.01). Peroxidase (POD) and catalase (CAT) activities were also positively correlated with multiple diversity indices (*r* = 0.61–0.73, *p* < 0.05). Conversely, malondialdehyde (MDA) content, an indicator of oxidative stress, exhibited negative correlations with Shannon (*r* = −0.65, *p* < 0.05) and Simpson indices (*r* = −0.66, *p* < 0.05), indicating reduced lipid peroxidation in high-diversity environments.

For photosynthetic pigments, total chlorophyll content showed moderate positive correlations with both Shannon (*r* = 0.58, *p* < 0.05) and Chao1 indices (*r* = 0.64, *p* < 0.05), suggesting that enhanced microbial diversity may promote chlorophyll accumulation. These results collectively demonstrate that soil microbial diversity is intimately associated with improved plant growth performance, enhanced antioxidant capacity, and maintained photosynthetic function. Given the small sample size (n = 3 per treatment), these correlation-based results are exploratory in nature and should be interpreted as hypothesis-generating rather than confirmatory. They serve to complement the primary physiological and biomass data presented above.

## 4. Discussion

### 4.1. Dose-Dependent Biomass Response and Resource Allocation Strategies

The biomass response of *P. capitata* to increasing PE-MP concentrations exhibited a typical hormetic pattern characterized by low-dose stimulation and high-dose inhibition [41,42]. This non-linear response has been previously documented in major crops such as maize and wheat [43,44], and here we report this phenomenon for the first time in the medicinal plant *P. capitata*. The mild stimulatory effect observed at the lowest concentration likely reflects an adaptive priming response. Under this scenario, low-level stress activates regulatory networks that transiently enhance photosynthetic efficiency or nutrient acquisition capacity [42]. The transition from stimulation to inhibition between the 1% and 4% treatments indicates the crossing of a toxicity threshold beyond which stress exceeds the plant compensatory capacity.

Notably, a significant increase in the root-to-shoot ratio occurred only under the highest concentration. This finding reveals a fundamental shift in resource allocation strategy under extreme stress. This morphological adjustment suggests that *P. capitata* adopts a conservative survival strategy. The plant prioritizes the allocation of limited photosynthates to root systems to maintain basic nutrient and water uptake capacity at the cost of aboveground growth [45]. While such plasticity is a common stress avoidance mechanism in plants, the long-term ecological costs of this reallocation deserve further investigation. These costs may include delayed flowering and reduced reproductive investment under microplastic pollution.

### 4.2. Pigment-Specific Responses and Microbially Mediated Nutrient Feedback

Changes in photosynthetic pigment content further corroborated the low-concentration stimulatory effect. The selective increase in chlorophyll a occurred without concurrent changes in chlorophyll b or the chlorophyll a/b ratio. This pattern indicates that PE-MP stress primarily affected the photosynthetic reaction center, which is dominated by chlorophyll a, while the light-harvesting antenna complex dominated by chlorophyll b remained relatively stable [46,47]. This differential sensitivity may arise from two complementary mechanisms. First, mild oxidative stress induced by low microplastic concentrations can upregulate key enzymes in chlorophyll synthesis such as glutamyl-tRNA reductase. This upregulation represents a compensatory protective response [48]. Second, the microbial data revealed a significant positive correlation between total chlorophyll content and rhizosphere microbial diversity based on Mantel tests. At the lowest concentration, microbial diversity indices remained statistically comparable to the control (Figure 4a,b), suggesting that the rhizosphere bacterial community was not substantially altered. Under this condition, the community likely maintained its capacity for nutrient mineralization, thereby indirectly supporting chlorophyll synthesis [49,50]. In contrast, under higher concentrations, the loss of microbial diversity particularly affected richness estimators such as Chao1 and ACE. This diversity loss may have weakened the supply of elements essential for chlorophyll synthesis including iron and magnesium, thereby impairing pigment biosynthesis [51,52].

This microbe–pigment link points to a broader nutrient-mediated cascade. Although soil nutrient availability was not quantified in the present experiment, previous studies have shown that high-dose PE-MP exposure can reduce soil available nitrogen and phosphorus by altering microbial mineralization activity and enhancing nutrient immobilization on microplastic surfaces [23,44]. This microbe–nutrition–photosynthesis cascade offers a novel mechanistic perspective. Microplastics may indirectly interfere with key plant metabolic pathways by disrupting soil microbial functional groups [53].

### 4.3. Synergistic Indicative Role of POD Inhibition and MDA Accumulation

Oxidative damage induced by PE-MPs followed a clear threshold pattern. Evidence of membrane lipid peroxidation reflected by elevated MDA content emerged only at the highest concentration. This finding indicates that high-dose microplastic exposure overwhelms membrane structural integrity [54]. However, the antioxidant enzyme response displayed a more complex and decoupled pattern. SOD activity remained stable across treatments. POD was significantly suppressed at low and medium concentrations. CAT showed a non-significant declining trend.

These decoupling warrants mechanistic consideration. Several factors may explain the inhibition of POD and CAT activities. First, microplastics or their leached additives such as phthalate plasticizers may directly bind to the iron porphyrin prosthetic groups in the active centers of these enzymes. Alternatively, these substances may interact with specific amino acid residues, inducing conformational changes and catalytic loss [55]. Second, the flux of reactive oxygen species induced by microplastics may exceed the regeneration capacity of the antioxidant system. This imbalance could lead to oxidative degradation of the enzyme proteins themselves [56]. Third, pollutants adsorbed onto microplastic surfaces or released metal ions may interfere with the biosynthesis of these iron-containing enzymes [57].

Critically, POD activity was significantly suppressed even at the lowest concentration, yet MDA did not increase correspondingly. This finding suggests that under low-concentration stress, plants may activate non-enzymatic antioxidant systems such as the ascorbate-glutathione cycle. These systems can scavenge reactive oxygen species and temporarily maintain redox homeostasis [58,59]. Only at the highest concentration are these compensatory mechanisms likely exhausted, resulting in widespread membrane lipid peroxidation. Collectively, compared to SOD or CAT, the early inhibition of POD and the late accumulation of MDA may serve as more sensitive indicators of PE-MP-induced oxidative stress progression [60,61].

The concurrent increase in root-to-shoot ratio under the highest concentration further supports the notion of a physical stress component (Figure 1c). Such shifts in resource allocation may reflect compensatory responses to physical impedance or epidermal clogging by microplastic particles. Previous research has reported that these physical interactions can impair water and nutrient uptake and trigger secondary oxidative stress [18,20]. Although soil physical properties were not directly quantified here, previous research indicates that microplastics can alter soil porosity and aggregate stability at concentrations comparable to our medium and high treatments [21,22]. Therefore, physical modifications represent a plausible contributor to the observed stress responses. The overall pattern, characterized by stable SOD and CAT, suppressed POD, and threshold-dependent MDA accumulation, underscores that the enzymatic antioxidant response in *P. capitata* is not uniformly activated under microplastic stress. This finding is consistent with previous reports of species-dependent and context-dependent antioxidant responses [16,17,35].

### 4.4. Loss of Microbial Diversity as a Key Mediator of Plant Growth Inhibition

Rhizosphere microbial community analysis provided critical microecological insight into the mechanisms underlying plant growth inhibition. PE-MPs particularly at medium concentrations significantly reduced bacterial richness as measured by Chao1 and ACE indices and reduced diversity as measured by the Shannon index. In contrast, the Simpson index remained unchanged. This pattern indicates that microplastics primarily diminished the richness of rare species with less impact on the structure of dominant populations [22,62]. This finding aligns with reports of microplastic-induced microbial community homogenization [63,64].

More critically, Mantel tests and correlation analyses revealed for the first time in *P. capitata* that microbial diversity loss was tightly coupled with inhibited plant growth, disturbed antioxidant enzyme activities, and exacerbated oxidative damage. This finding strongly supports our core argument. PE-MPs harm plants not only through direct physicochemical pathways but also, and perhaps more importantly, by disrupting the stability and functional redundancy of the rhizosphere microecosystem. Such disruption may sever mutually beneficial interactions between plants and beneficial microorganisms, including phosphate-solubilizing bacteria and siderophore-producing bacteria, thereby amplifying stress effects [65]. The significant negative correlation between the root-to-shoot ratio and the Shannon index further suggests that when microbial diversity declines and the soil environment deteriorate, plants are forced to invest more resources into root systems. This investment compensates for the loss of functional microorganisms but incurs substantial adaptive costs [66,67].

In addition to microbial shifts, the physical presence of microplastic particles may contribute to growth inhibition through alterations in soil physical properties. Although not directly measured here, previous studies have shown that microplastics can modify bulk density and water-holding capacity, indirectly affecting root growth and nutrient acquisition [21,22,28]. Thus, the observed growth inhibition at higher concentrations likely involves a combination of direct oxidative stress, microbial community disruption, and physical modifications to the root microenvironment.

Whether these PE-MP-induced shifts in the rhizosphere microbiome are reversible upon removal of the microplastic source remains an open question, as direct empirical evidence on microbial recovery following microplastic cessation is currently limited [1]. Several lines of evidence from this study suggest that recovery may be constrained. First, the significant reduction in bacterial richness (Chao1 and ACE indices) under medium and high PE-MP concentrations indicates that sensitive taxa, particularly rare species, may have been selectively lost. Such diversity loss often exhibits hysteresis, meaning that community recovery may be slow or incomplete even after stressor removal due to the extinction of keystone taxa or the establishment of alternative stable states [22]. Second, the tight coupling between microbial diversity loss and plant growth inhibition (Figure 5) implies a feedback mechanism: impaired root exudation under stress could limit carbon inputs to the rhizosphere, thereby hindering microbial recolonization even after microplastic removal [68]. Conversely, the stability of the Simpson index—reflecting the persistence of dominant taxa—suggests that functional redundancy may allow maintenance of key ecosystem processes despite taxonomic shifts [69].

### 4.5. Research Limitations and Future Perspectives

While this study provides valuable insights into the dose-dependent effects of PE-MPs on *P. capitata*, several limitations should be acknowledged. First, while growth parameters were quantified using conventional biomass measurements (shoot and root fresh weight), independent bioassays such as seed germination tests or root elongation assays were not conducted. Second, this study did not quantify pharmacologically active secondary metabolites (e.g., gallic acid, quercetin) in *P. capitata*, leaving its therapeutic viability under PE-MP stress unknown. Third, the specific microbial taxa driving rhizosphere bacterial diversity shifts under PE-MP stress were not identified. Whether diversity loss reflects depletion of beneficial taxa or enrichment of stress-tolerant taxa remains unresolved. Fourth, soil physicochemical properties including bulk density, pH, organic matter content, and nutrient availability were not directly measured. Mechanistic inferences involving soil physical and chemical pathways therefore remain supported primarily by indirect evidence. Fifth, the limited replication (n = 3) restricts statistical power for multivariate analyses, precluding strong causal inference from the exploratory correlation patterns observed.

To address these limitations, future research should prioritize the following directions: (1) Incorporate standardized bioassays, such as seed germination tests and root elongation assays, to provide complementary insights into the early toxicological responses of plants to microplastic exposure and validate the dose-dependent effects observed here. (2) Conduct metabolomic analyses to assess whether secondary metabolites follow similar concentration-dependent patterns as growth and chlorophyll responses, and to evaluate medicinal output per unit land area under PE-MP stress. (3) Employ 16S rRNA sequencing with taxonomic assignment and root exudate metabolomics to identify key microbial taxa and disentangle direct and indirect mechanisms underlying community responses to PE-MP stress. (4) Integrate comprehensive soil physicochemical analyses, including bulk density, pH, organic matter content, and nutrient availability, into experimental designs to enable direct quantification of microplastic impacts on soil–plant systems. (5) Increase experimental replication to enhance statistical power, enabling more robust assessment of relationships between microplastic exposure, rhizosphere microbiomes, and plant physiological responses. Such integrated approaches are essential for establishing mechanistic links between microplastic transformation pathways and their cascading effects on soil–plant systems.

## 5. Conclusions

This study demonstrates that polyethylene microplastics (PE-MPs) exert dose-dependent effects on *P. capitata*, with a hormetic response characterized by growth stimulation at 1% (*w*/*w*) but significant inhibition at 4% and 8%. Chlorophyll a was more sensitive to microplastic stress than chlorophyll b, and its decline at high concentrations was closely associated with reduced rhizosphere bacterial diversity. Within the antioxidant system, peroxidase (POD) activity was significantly suppressed at low and medium concentrations. In contrast, malondialdehyde (MDA) content, which serves as a marker of lipid peroxidation, increased significantly under the 8% treatment only. Superoxide dismutase and catalase showed no significant changes. These results indicate that POD and MDA served as more sensitive indicators of oxidative damage under PE-MP exposure. Critically, PE-MPs significantly reduced the richness and diversity of rhizosphere bacterial communities, and the loss of microbial diversity was closely associated with inhibited plant growth and exacerbated oxidative damage. These findings suggest that rhizosphere microecological imbalance may represent an important indirect mechanism underlying microplastic-induced phytotoxicity, although further studies with larger sample sizes and mechanistic approaches are needed to substantiate this interpretation. This study provides the first evidence of microplastic-induced ecological effects on the medicinal plant *P. capitata* and elucidates the underlying microecological mechanisms, offering a theoretical basis for soil health assessment in karst regions and highlighting the necessity of integrating microbial community dynamics into phytotoxicity risk evaluations.

## Data Availability

The original contributions presented in this study are included in this article. Further inquiries can be directed to the corresponding author.
